# Food Rather Than Physic

**Published:** 1867-12

**Authors:** 


FOOD RATHER THAN PHYSIC.

As evidence of its capacity to fatten children a physician states in the American Journal of Pharmacy, its chemical formula being six parts carbon, seven parts hydrogen and five parts oxygen,

1st. An infant six months old, recovering from a severe diarrhœa, kept quite emanciated and pale. Glycerine was ordered for it, and in a few days a change was remarked in its appearance for the better, and in four weeks it weighed eight pounds heavier. 2. A child, sixteen months old, had its head covered with one continous scab,—porrigo. This was a family complaint, and resisted all manner of treatment for a very long time in all the other children. In the above case I resorted to glycerine, both internally and externally.

A cure was effected in three months. 3. A girl, seven years old, recovering from measles, retained her cough, emaciation, and nervous irritability. Dullness over apex of left lung; roughened breathing. No doubt the case was chronic pneumonia. Glycerine, as a last resort, was ordered in teaspoonful doses in water, three times a day. Recovery in six weeks. 4. A strumous boy, much emaciated, had hacking cough and night sweats. Pulse frequent. Sleep disturbed. Abdomen tumid and enlarged. Cervical glands swollen. Bowels irregular. Fecal discharges clay-colored. His case was such, that no one expected any more than a partial palliation. After other treatments had failed, I ordered glycerine in teaspoonful doses, in which were dissolved four grains of ferri ammon. cit., and one half of a grain of quinia, four times a day. This he continued for a year, and was in remarkably good health three years after.

